# Supplementary material for: Heat adaptation of phage T7 under an extended genetic code
Source: Virus Evol. 2021 Dec 1;7(2):veab100. doi: 10.1093/ve/veab100 (PMC8923235; doi:10.1093/ve/veab100)
Supplement: veab100_Supp [file veab100_supp.zip › Sequencing_Analysis.zip!/Sequencing_Analysis/S2/evidence/SNP_7.html]

BRESEQ :: Evidence


| Predicted mutation | | | | | | |
| --- | --- | --- | --- | --- | --- | --- |
| evidence | seq id | position | mutation | annotation | gene | description |
| RA | NC\_001604.1 | 21,285 | A→C | intergenic (–/–) | – / – | –/– |

| Read alignment evidence... | | | | | | | | | | | |
| --- | --- | --- | --- | --- | --- | --- | --- | --- | --- | --- | --- |
|  | seq id | position | | ref | new | freq | score (cons/poly) | reads | annotation | genes | product |
| \* | NC\_001604.1 | 21,285 | 0 | A | C | 89.9% | 13878.9 / inf | 5235 | intergenic (–/–) | *–/–* | –/– |
| Reads supporting (aligned to +/- strand):  **ref** base A (262/265);  **new** base C (2231/2457);  **total** (2505/2730) | | | | | | | | | | | |
| Fisher's exact test for biased strand distribution *p*-value = 3.58e-01 | | | | | | | | | | | |
| Kolmogorov-Smirnov test that lower quality scores support variant *p*-value = 8.47e-03 | | | | | | | | | | | |

Only 100 of 5236 total aligned reads displayed.  

`CCATACATCCCGATTCGGATGGTCAGACTAGATGGTGAATCCTACGGTCGTTCGTACATTGAGGAATACTTAGGTGACTTACGGTCCCTTGAAAATCTCCAAGAGGCTATCGTCAAGATGTCCATGATTAGCTCTAAGGTTATCGGCTTAGTGAATCCTGCTGGTATCACCCAGCCACGCCGACTGACCAAAGCTCAGACTGGTGACTTCGTTACTGGTCGTCCAGAAGACATCTCGTTCCTCCAACTGGAGAAGCAAGCAGACTTTACTGTAGCTAAAGCCGTAAGTGACGCTATCGAGGCTCGCCTTTCGTTTGCCTTTATGTTGAACTCTGCGGTTCAGCGTACAGGTGAACGTGTGACCGCCGAAGAGATTCGGTATGTAGCTTCTGAACTTGAAGATACTTTAGGTGG‑TGTCTACTCTATCCTTTCTCAAGAATTACAATTGCCTCTGGTACGAGTGCTCTTGAAGCAACTACAAGCCACGCAACAGATTCCTGAGTTACCTAAGGAAGCCGTAGAGCCAACCATTAGTACAGGTCTGGAAGCAATTGGTCGAGGACAAGACCTTGATAAGCTGGAGCGGTGTGTCACTGCG  >  NC_001604.1/20987‑21583`  

                                                                                                                                                                                                                                                                                                          `|`                                                                                                                                                                                                                                                                                                             
      `aTCCCGAGTCGGATGGTCAGACTAGATGGTGAATCCTACGGTCGTTCGTACATTGAGGATTACTTAGGTGACTTACGGTCCCTTGAAAATCTCCAAGATGCTATCGTCAAGATGTCCATGATTAGCTCTAAGGTTATCGGCTTAGTGAATCCTGCTGGTATCACCCAGCCACGCCGACTGACCAAAGCTCAGACTGGTGACTTCGTTACTGGTCGTCCAGAAGACATCTCGTTCCTCCAACTGGAGAAGCAAGCAGACTTTACTGTAGCTAACGCCGTAAGTGACGCTATCGCGGCTCGcc                                                                                                                                                                                                                                                                                                     <  1:360804/301‑1 (MQ=255)`  
                 `gggagatgtgtataagagacagtCCTACGGTCGTTCGTACATTGAGGAATACTTAGGTGACTTACGGTCCCTTGAAAATCTCCAAGATGCTATCGTCAAGATGTCCATGATTAGCTCTAAGGTTATCGGCTTAGTGAATCCTGCTGGTATCACCCAGCCACGCCGACTGACCAAAGCTCAGACTGGTGACTTCGTTACTGGTCGTCCAGAAGACATCTCGTTCCTCCAACTGGAGAAGCAAGCAGACTTTACTGTAGCTAACGCCGTAAGTGACGCTATCGCGGCTCGCCTTTCGTTTGcc                                                                                                                                                                                                                                                                                          <  1:449402/279‑1 (MQ=255)`  
                 `gATGGTCAGACTAGATGGTGAATCCTACGGTCGTTCGTACATTGAGGAATACTTAGGTGACTTACGGTCCCTTGAAAATCTCCAAGAGGCTATCGTCAAGATGTCCATGATTAGCTCTAAGGTTATCGGCTTAGTGAATCCTGCTGGTATCACCCAGCCACGCCGACTGACCAAAGCTCAGACTGGTGACTTCGTTACTGGTCGTCCAGAAGACATCTCGTTCCTCCAACTGGAGAAGCAAGCAGACTTTACTGTAGCTAACGCCGTAAGTGACGCTATCGCGGCTCGCCTTTCGTTTGcc                                                                                                                                                                                                                                                                                          <  1:465060/301‑1 (MQ=255)`  
                        `gacagaGATGGTGAATCCTACGGTCGTTCGTACATTGAGGAATACTTAGGTGACTTACGGTCCCTTGAAAATCTCCAAGAGGCTATCGTCAAGATGTCCATGATCAGCTCTAAGGTTATCGGCTTAGTGAATCCTGCTGGTATCACCCAGCCACGCCGACTTACCAAAGCTCAGACTGGTGACTTCGTTACTGGTCGTCCAGAAGACATCTCGTTCCTCCAACTGGAGAAGCAAGCAGACTTTACTGTAGCTAACGCCGTAAGTGACGCTATCGCGGCTCGCCTTTCGTTTGCCTTTATGt                                                                                                                                                                                                                                                                                   <  1:157532/296‑1 (MQ=255)`  
                         `gACTAGATGGTGAATCCTACGGTCGTTCGTACATTGAGGAATACTTAGGTGACTTACGGTCCCTTGAAAATCTCCAAGAGGCTATCGTCAAGATGTCCATGATTAGCTCTAAGGTTATTGGCTTAGTGAATCCTGCTGGTATCACCCAGCCACGCCGACTGACCAAAGCTCAGACTGGTGACTTCGTTACTGGTCGTCCAGAAGACATCTCGTTCCTCAAACTGGAGAAGCAAGCAGACTTTACTGTAGCTAACGCCTTAAGTGACGCTATTGCCGCCCGCCTTTCGTTGGCCtttttctt                                                                                                                                                                                                                                                                                  >  1:405084/1‑296 (MQ=255)`  
                               `aTGGTGAATCCTACGGTCGTTCGTACATTGAGGAATACTTAGGTGACTTACGGTCCCTTGAAAATCTCCAAGAGGCTATCGTCAAGATGTCCATGATTAGCTCTAAGGTTATCGGCTTAGTGAATCCTGCTGGTATCGCCCAGCCACGCCGACTGACCAAAGCTCAGACTGGTGACTTCGTTACTGGTCGTCCAGAAGACATCTCGTTCCTCCAACTGGAGAAGCAAGCAGACTTTACTGTAGCTAACGCCGTAAGTGACGCTATCGCGGCTCGCCTTTCGTTTGCCTTTATGTTGAAcct                                                                                                                                                                                                                                                                            >  1:345035/1‑299 (MQ=255)`  
                                 `ggTGAATCCTACGGTCGTTCGTACATTGAGGAATACTTAGGTGACTTACGGTCCCTTGAAAATCTCCAAGAGGCTATCGTCAAGATGTCCATGATTAGCTCTAAGGTTATCGGCTTAGTGAATCCTGCTGGTATCACCCAGCCACGCCGACTGACCAAAGCTCAGACTGGTGACTTCGTTACTGGTCGTCCAGAAGACATCTCGTTCCTCCAACTGGAGAAGCAAGCAGACTTTACTGTAGCTAACGCCGTAAGTGACGCTATCGCGGCTCGCCTTTCGTTTGCCTTTATGTTGCActctt                                                                                                                                                                                                                                                                          >  1:368534/1‑300 (MQ=255)`  
                                 `ggTGAATCCTACGGTCGTTCGTACATTGAGGAATACTTAGGTGACTTACGGTCCCTTGAAAATCTCCAAGAGGCTATCGTCAAGATGTCCATGATTAGCTCTAAGGTTATCGGCTTAGTGAATCCTGCTGGTATCACCCAGCCACGCCGACTGACCAAAGCTCAGACTGGTGACTTCGTTACTGGTCGTCCAGAAGACATCTCGTTCCTCCAACTGGAGAAGCAAGCAGACTTTACTGTAGCTAACGCCGTAAGTGACGCTATCGCGGCCCGCCTTTCGTTTGCCTTTATGTTGAAcctgt                                                                                                                                                                                                                                                                          >  1:187948/1‑297 (MQ=255)`  
                                      `aTCCTACGGTCGTTCGTACATTGAGGAATACTTAGGTGACTTACGGTCCCTTGAAAATCTCCAAGAGGCTATCGTCAAGATGTCCATGATTAGCTCTAAGGTTATCGGCTTAGTGAATCCTGCTGGTATCACCCAGCCACGCCGACTGACCAAAGCTCAGACTGGTGACTTCGTTACTGGTCGTCCAGAAGACATCTCGTTCCTCCAACTGGAGAAGCAAGCAGACTTTACTGTAGCTAACGCCGTAAGTGACGCTATCGCGGCTCGCCTTTCGTTTGCCTTTATGTTGAACTCTTCGGtt                                                                                                                                                                                                                                                                     >  1:471144/1‑301 (MQ=255)`  
                                       `tCCTACGGTCGTTCGTACATTGAGGAATACTTAGGTGACTTACGGTCCCTTGAAAATCTCCAAGAGGCTATCGTCAAGATGTCCATGATTAGCTCTAAGGTTATCGGCTTAGTGAATCCTGCTGGTATCACCCAGCCACTCCGACTGACCAAAGCTCAGACTGGTGACTTCGTTACTGGTCGTCCAGAAGACATCTCGTTCCTCCAACTGGAGAAGCAAGCAGACTTTACTGTAGCTAACGCCGTAAGTGACGCTATCGCGGCTCGCCTTTCGTTTGCCTTTATGTTGAACTCTGCGGTTc                                                                                                                                                                                                                                                                    >  1:233201/1‑301 (MQ=255)`  
                                         `aagagacagttcgtACATTGAGGAATACTTAGGTGACTTACGGTCCCTTGAAAATCTCCAAGAGGCTATCGTCAAGATGTCCATGATTAGCTCTAAGGTTATCGGCTTAGTGAATCCTGCTGGTATCACCCAGCCACGCCGACTGACCAAAGCTCAGACTGGTGACTTCGTTACTGGTCGTCCAGAAGACATCTCGTTCCTCCAACTGGAGAAGCAAGCAGACTTTACTGTAGCTAACGCCGTAAGTGACGCTATCGCGGCTCGCCTTTCGTTTGCCTTTATGTTGAACTCTGCGGTTCAg                                                                                                                                                                                                                                                                  <  1:530963/293‑1 (MQ=255)`  
                                            `ggagatgtgtataagagacagaTACTTAGGTGACTTACGGTCCCTTGAAAATCTCCAAGAGGCTATCGTCAAGATGTCCATGATTAGCTCTAAGGTTATCGGCTTAGTGAATCCTGCTGGTATCACCCAGCCACGCCGACTGACCAAAGCTCAGACTGGTGACTTCGTTACTGGTCGTCCAGAAGACATCTCGTTCCTCCAACTGGAGAAGCAAGCAGACTTTACTGTAGCTAACGCCGTAAGTGACGCTATCGCGGCTCGCCTTTCGTTTGCCTTTATGTTGAACTCTGCGGTTCAGCGt                                                                                                                                                                                                                                                               <  1:85572/280‑1 (MQ=255)`  
                                            `cGGTCGTTCGTACATTGAGGAATACTTAGGTGACTTACGGTCCCTTGAAAATCTCCAAGAGGCTATCGTCAAGATGTCCATGATTAGCTCTAAGGTTATCGGCTTAGTGAATCCTGCTGGTATCACCCAGCCACGCCGACTGACCAAAGCTCAGACTGGTGACTTCGTTACTGGTCGTCCAGAAGACATCTCGTTCCTCCAACTGGAGAAGCAAGCAGACTTTACTGTAGCTAACGCCGTAAGTGACGCTATCGCGGCTCGCCTTTCGTTTGCCTTTATGTTGAACTCTGCGGTTCAGCGt                                                                                                                                                                                                                                                               <  1:92520/301‑1 (MQ=255)`  
                                                 `ataagagacaggAGGAATACTTAGGTGACTTACGGTCCCTTGAAAATCTCCAAGAGGCTATCGTCAAGATGTCCATGATTAGCTCTAAGGTTATCGGCTTAGTGAATCCTGCTGGTATCACCCAGCCACGCCGACTGTCCAAAGCTCAGACTGGTGACTTCGTTACTGGTCGTCCAGAAGACATCTCGTTCCTCCAACTGGAGAAGCAAGCAGACTTTACTGTAGCTAACGCCGTAAGTGACGCTATCGCGGCTCGCCTTTCGTTTGCCTTTATGTTGAACTCTGCGGTTCAGCGTACAgg                                                                                                                                                                                                                                                          <  1:201341/290‑1 (MQ=255)`  
                                                  `ttcgtACATTGAGGAATACTTAGGTGACTTACGGTCCCTTGAAAATCTCCAAGAGGCTATCGTCAAGATGTCCATGATTAGCTCTAAGGTTATCGGCTTAGTGAATCCTGCTGGTATCACCCAGCCACGCCGACTGACCAAAGCTCAGACTGGTGACTTCGTTACTGGTCGTCCAGAAGACATCTCGTTCCTCCAACTGGAGAAGCAAGCAGACTTTACTGTAGCTAACGCCGTAAGTGCCGCTATCGAGGCTCGCCTTTCGTTTGCCTTTATGTTGAAcctgtctcttatacacatctcc                                                                                                                                                                                                                                                         >  1:809296/1‑280 (MQ=255)`  
                                                  `ttcgtACATTGAGGAATACTTAGGTGACTTACGGTCCCTTGAAAATCTCCAAGAGGCTATCGTCAAGATGTCCATGATTAGCTCTAAGGTTATCGGCTTAGTGAATCCTGCTGGTATCACCCAGCCACGACGACTGAACAAAGCTCAGACTGGTGACTTCGTTACTGGTCGTCCAGAAGACATCTCGTTCCTCCAACTGGAGAAGCAAGCAGACTTTACTGTAGCTAACGCCGTAAGTGACGCTATCGCGGCTCGCCTTTCGTTTGCCTTTATGTTGAACTCTGcctgtctcttatacaca                                                                                                                                                                                                                                                         >  1:420676/1‑285 (MQ=255)`  
                                                  `ttcgtACATTGAGGAATACTTAGGTGACTTACGGTCCCTTGAAAATCTCCAAGAGGCTATCGTCAAGATGTCCATGATTAGCTCTAAGGGTATCGGCTTAGTGAATCCTGCTGGTATCACCCAGCCACGCCGACTGACCAAAGCTAAAACTGGTGCAGTCGTTACTGGTCGTCCAGAAGACAGCTCGTTCCTCCAACTGGAGAAGCAAGCAGACTGTACTGTAGCTAACGACGTAAGTGACGCTATCGCGGCTCGCCTTTCGTTTGCCTCTATGTTGAACTCTGCGGTTCAGCGTATAGGt                                                                                                                                                                                                                                                         >  1:70201/1‑301 (MQ=255)`  
                                                     `gtACATTGAGGAATACTTATGTGACTTACGGTCCCTTGAAAATCTCCAAGAGGCTATCGTCAAGATGTCCATGATTAGCTCTAAGGTTATCGGCTTAGTGAATCCTGCTGGTATCACCCAGCCACGCCGACTGACCAAAGCTCAGACTGGTGACTTCGTTACTGGTCGTCCAGAAGACATCTCGTTCCTCCAACTGGAGAAGCAAGCCGACTTTACTGTAGCTAACGCCGTAAGTGACGCTATCGCGGCTCGCCTTTCGTTTGCCTTTATGTTGAACTCTGCGGTTCAGCGTACAGGTGaa                                                                                                                                                                                                                                                      >  1:390742/1‑301 (MQ=255)`  
                                                         `aTTGAGGAGTAATTAGGTGACTTACGGTCACTAGAAAATCTCCAAGCGGCTATCGTAAAGATGTCCATGATTAGCTCTAAGGTTATCGGCTTAGTGAATCCAGCTGGTCTAACCCAGCCAAGCCGACTGACCAAAGCTTATACTGGTGACTTAGTTACTGGTCGTCCAGAAGCCTTATTGTTCCTCCAACTGGAGAAGCCAGCCGACTTTACTGTAGCTACAGCCGTAAGTCACGCTAACGCAGCACGCCTTGCGTTCGCCTTTCTGTTGAGCGCTGGGGTTCAGCTTACGGAGGAACgtg                                                                                                                                                                                                                                                  >  1:128879/1‑301 (MQ=255)`  
                                                          `ttGAGGAATACTTAGGTGACTTACGGTCCCTTGAAAATCTCCAAGAGGCTATCGTCAAGATGTCCATGATTAGCTCTAAGGTTATCGGCTTAGTGAATCCTGCTGGTATCACCCAGCCACGCCGACTGACCAAAGCTCAGACTGGTGACTTCGTTACTGGTCGTCCAGAAGACATCTCGTTCCTCCAACTGGAGAAGCAAGCAGACTTTACTGTAGCTAACGCCGTAAGTGACGCTATCGCGGCTCGCCTTTCGTTTGCCTTTATGTTGAACTCTGCGGTTCAGCGTAcctgtctcttata                                                                                                                                                                                                                                                 >  1:508964/1‑289 (MQ=255)`  
                                                           `tGAGGAATACTTAGGTAACTTACGGTCACTTGAAAATATCCAAGAGGCTATCGTCAAGATGTCCATGATTAGCTCTAAGGTTATCGGCTTAGTGAATCCTGCTGGTATCACCCAGCCACGCCGACTGACCAAAGCTCAGACTGGTGACTTAGTTACTGGTCGTCCAGAAGACATCTCGTTCCTCCAACTGGAGAAGCAAGCAGACTTTACTGTAGCTAACGCCGTAAGTGCCGCTATCGAGGATCGCCTTTCGTTTGCCTTTATGTTGAACTCTGCGGTTCAGCGTACAGGTGAACGtgtg                                                                                                                                                                                                                                                <  1:165070/301‑1 (MQ=255)`  
                                                                 `aTACTTAGGTGACTTACGGTCCCTTGAAAATCTCCAAGAGGCTATCGTCAAGATGTCCATGATTAGCTCTAAGGTTATCGGCTTAGTGAATCCTGCTGGTATCACCCAGCCACGCCGACTGACCAAAGCTCAGACTGGTGACTTCGTTACTGGTCGTCCAGAAGACATCTCGTTCCTCCAACTGGAGAAGCAAGCAGACTTTACTGTAGCTAACGCCGTAAGTGACGCTATCGCGGCTCGCCTTTCGTTTGCCTTTATGTTGAACTCTGCGGTTCAGCGTACAGGTGAACGtgtgctgtct                                                                                                                                                                                                                                          >  1:516256/1‑295 (MQ=255)`  
                                                                   `aCTTAGGTGACTTACGGTCCCTTGAAAATCTCCAAGAGGCTATCGTCAAGATGTCCATGATTAGCTCTAAGGTTATCGGCTTAGTGAATCCTGCTGGTATCACCCAGCCACGCCGACTGACCAAAGCTCAGACTGGTGACTTCGTTACTGGTCGTCCAGGAGACATCTCGTTCCTCCAACTGGAGAAGCAAGCAGACTTTACTGTAGCTAGCGCCGTAAGTGACGCTATCGCGGCTCGCCTTTCGTTTGCCTTTATGTTGAACTCTGCGGTTCAGCGTACAGGTGAACGTGTGACCGCCGa                                                                                                                                                                                                                                        >  1:100257/1‑301 (MQ=255)`  
                                                                       `aGGTGACTTACGGTCCCTTGAAAATCTCCAAGAGGCTATCGTCAAGATGTCCATGATTAGCTCTAAGGTTATCGGCTTAGTGAATCCTGCTGGTATCACCCAGCCACGCCGACTGACCAAAGCTCAGACTGGTGACTTCGTTACTGGTCGTCCAGAAGACATCTCGTTCCTCCAACTGGAGAAGCAAGCAGACTTTACTGTAGCTAACGCCGTAAGTGCCGCTATCGAGGCTCGCCTTTCGTTTGCCTTTATGTTGAACTCTGCGGTTCAGCGTACAGGTGAACGTGTGACCGCCGAagag                                                                                                                                                                                                                                    <  1:694598/301‑1 (MQ=255)`  
                                                                          `tGACTTACGGTCCCTTGAAAATCTCCAAGAGGCTATCGTCAAGATGTCCATGATTAGCTCTAAGGTTATCGGCTTAGTGAATCCTGCTGGTATCACCCAGCCACGCCGACTGACCAAAGCTCAGACTGGTGACTTCGTTACTGGTCGTCCAGAAGACATCTCGTTCCTCCAACTGGAGAAGCAAGCAGACTTTACTGTAGCTAACGCCGTAAGTGACGCTATCGCGGCTCGCCTTTCGTTTGCCTTTATGTTGAACTCTGCGGTTCAGCGTACAGGTGAACGTGTGACCGCCGAAGAGAtt                                                                                                                                                                                                                                 <  1:500847/301‑1 (MQ=255)`  
                                                                           `gACTTACGGTCCCTTGAAAATCTCCAAGAGGCTATCGTCAAGATGTCCATGATTAGCTCTAAGGTTATCGGCTTAGTGAATCCTGCTGGTATCACCCAGCCACTCCGACTGACCAAAGCTCAGACTGGTGACTTCGTTACTGGTCGTCCAGAAGACATCTCGTTCCTCCAACTGGAGAAGCAAGCAGACTTTACTGTAGCTAACGCCGTAAGTGACGCTATCGCGGCTCGCCTTTCGTTTTCCTTTATGTTGAACTCTGCGGTTCAGCGTACAGGTGAACGTGTGACCGCCGAAGAGATTc                                                                                                                                                                                                                                >  1:591336/1‑301 (MQ=255)`  
                                                                             `cTTACGGTCCCTTGAAAATCTCCAAGAGGCTATCGTCAAGATGTCCATGATTAGCTCTAAGGTTATCGGCTTAGTGAATCCTGCTGGTATCACCCAGCCACGCCGACTGACCAAAGCTCAGACTGGTTACTTCGTTACTGGTCGTCCAGAAGACATCTCGTTCCTCCAACTGGAGAAGCAAGCAGACTTTACTGTAGCTAACGCCGTAAGTGACGCTATCGCGGCTCGCCTTTCGTTTGCCTTTATGTTGAACTCTGCGGTTCAGCGTACAggctgtctcttatacacatctccgagcccc                                                                                                                                                                                                                              >  1:559499/1‑273 (MQ=255)`  
                                                                              `ttACGGTCCCTTGAAAATCTCCAAGAGGCTATCGTCAAGATGTCCATGATTAGCTCTAAGGTTATCGGCTTAGTGAATCCTGCTGGTATCACCCAGCCACGCCGACTGACCAAAGCTCAGACTGGTGACTTCGTTACTGGTCGTCCAGAAGACATCTCGTTCCTCCAACTGGAGAAGCAAGCAGACTTTACTGTAGCTAACGCCGTAAGTGACGCTATCGCGGCTCGCCTTTCGTTTGCCTTTATGTTGAACTCTGCGGTTCAGCGTACAGGTGAACGTGTGACCGCCGAAGAGATTCGGt                                                                                                                                                                                                                             >  1:427438/1‑301 (MQ=255)`  
                                                                                    `gacagggAAAATCTCCAAGAGGCTATCGTCAAGATGTCCATGATTAGCTCTAAGGTTATCGGCTTAGTGAATCCTGCTGGTATCACCCAGCCACGCCGACTGACCAAAGCTCAGACTGGTGACTTCGTTACTGGTCGTCCAGAAGACATCTCGTTCCTCCAACTGGAGAAGCAAGCAGACTTTACTGTAGCTAACGCCGTAAGTGACGCTATCGCGGCTCGCCTTTCGTTTGCCTTTATGTTGAACTCTGCGGTTCAGCGTACAGGTGAACGTGTGACCGCCGAAGAGATTCGGTATGTAg                                                                                                                                                                                                                       <  1:180657/295‑1 (MQ=255)`  
                                                                                             `aaTCTCCAAGAGGCTATCGTCAAGATGTCCATGATTAGCTCTAAGGTTATCGGCTTAGTGAATCCTGCTGGTATCACCCAGCCACGCCGACTGACCAAAGCTCAGACTGGTGACTTCGTTACTGGTCGTCCAGAAGACATCTCGTTCCTCCAACTGGAGAAGCAAGCAGACTTTACTGTAGCTAACGCCGTAAGTGACGCTATCGCGGCTCGCCTTTCGTTTGCCTTTATGTTGAACTCTGCGGTTCAGCGTACAGGTGAACGTGTGACCGCCGAAGAGATTCGGTATGTAGCTTCTGAAc                                                                                                                                                                                                              <  1:715467/301‑1 (MQ=255)`  
                                                                                                        `ggCTATCGTCAAGATGTCCATGATTAGCTCTAAGGTTATCGGCTTAGTGAATCCTGCTGGTATAACCCAGCCACGCCGACTGACCAAAGCTCAGACTGGTGACTTCGTTACTGGTCGTCCAGAAGACATCTCGTTCCTCCAACTGGAGAAGCAAGCAGACTTTACTGTAGCTAACGCCGTAAGTGACGCTATCGCGGCTCGCCTTTCGTTTGCCTTTATGTTGAACTCTTCGGTTCAGCGTACAGGTGAACGTGTGACCGCCGAAGAGATTCGGTATGTAGCTCCTGAACTTGAAGATACt                                                                                                                                                                                                   >  1:655009/1‑301 (MQ=255)`  
                                                                                                         `gCTATCGTCAAGATGTCCATGATTAGCTCTAAGGTTATCGGCTTAGTGAATCCTGCTGGTATCACCCAGCCACGCCGACTGACAAAAGCTCAGACTGGTGACTTCGTTACTGGTCGTCCAGAAGACATCTCGTTCCTCCAACTGGAGAAGCAAGCAGACTTTACTGTAGCTAACGCCGTAAGTGACGCTATCGCGGCTCGCCTTTCGTTTGCCTTTATGTTGAACTCTGCGGTTCAGCGTACAGGTGAACGTGTGACCGCCGAAGGGCTTCGGTATGTAGCTTCTGAACTTGAAGATCCtt                                                                                                                                                                                                  >  1:258676/1‑301 (MQ=255)`  
                                                                                                            `aTCGTCAAGATGTCCATGATTAGCTCTAAGGTTATCGGCTTAGTGAATCCTGCTGGTATCACCCAGCCACGCCGACTGACCAAAGCTCAGACTGGTGACTTCGTTACTGGTCGTCCAGAAGACATCTCGTTCCTCCAACTGGAGAAGCAAGCAGACTTTACTGTAGCTAACGCCGTAAGTGACGCTATCGCGGCTCGCCTTTCGTTTGCCTTTATGTTGAACTCTGCGGTTCAGCGTACAGGTGAACGTGTGACCGCCGAAGAGATTCGGTATGTAGCTTCTGAACTTGAAGATACTTTAg                                                                                                                                                                                               >  1:484230/1‑301 (MQ=255)`  
                                                                                                               `gTCAAGATGTCCATGATTAGCTCTAAGGTTATCGGCTTAGTGAATCCTGCTGGTATCACCCAGCCACGCCGACTGACCAAAGCTCAGACTGGTGACTTCGTTACTGGTCGTCCAGAAGACATCTCGTTCCTCAAACTGGAGAAGCAAGCAGACTTTACTGTAGCTAACGCCGTAAGTGACGCTATCGCGGCTCGCCTTTCGTTTGCCTTTATGTTGACCTCTGCGGTTCAGCGTACAGGTGAACGTGTGACCGCCGAAGAGATCCGGTATGTAGCTTCTGAACTTGAAGATACTTTAggtg                                                                                                                                                                                            >  1:382614/1‑301 (MQ=255)`  
                                                                                                                `taaaGATGTCCATGATTAGCTCTAAGGTTATCGGCTTAGTGAATCCTGCTGGTATCACCCAGCCACGCCGACTGACCAAAGCTCAGACTGGTGACTTCGTTACTGGTCGTCCAGAAGACATCTCGTTCCTCCAACTGGAGAAGCAAGCAGACTTTACTGTAGCTAACGCCGTAAGTGACGCTATCGCGGCTCGCCTTTCGTTTGCCTTTATGTTGAACTCTGCGGTTCAGCGTACAGGTGAACGTGTGACCGCCGAAGAGATTCGGTATGTAGCTTCTGAACTTGAAGATACTTTAggtgg                                                                                                                                                                                           <  1:583118/299‑1 (MQ=255)`  
                                                                                                                `tCAAGATGTCCATGATTAGCTCTAAGGTTATCGGCTTAGTGAATCCTGCTGGTATCACCCAGCCACGCCGACTGACCAAAGCTCAGACTGGTGACTTCGTTACTGGTCGTCCAGAAGACATCTCGTTCCTCCAACTGGAGAAGCAAGCAGACTTTACTGTAGCTAACGCCGTAAGTGACGCTATCGCGGCTCGCCTTTCGTTTGCCTTTATGTTGAACTCTGCGGTTCAGCGTACAGGTGAACGTGTGACCGCCGAAGAGATTCGGTATGTAGCTTCTGAACTTGAAGATACTTTAggtgg                                                                                                                                                                                           <  1:398229/301‑1 (MQ=255)`  
                                                                                                                       `gTCCATGATTAGCTCTAAGGTTATCGGCTTAGTGAATCCTGCTGGTATCACCCAGCCACGCCGACTGACCAAAGCTCAGACTGGTGACTTCGTTACTGGTCGTCCAGAAGACATCTCGTTCCTCCAACTGGAGAAGCAAGCAGACTTTACTGTAGCTAACGCCGTAAGTGACGCTATCGCGGCTCGCCTTTCGTTTGCCTTTATGTTGAACTCTGCGGTTCAGCGTACAGGTGAACGTGTGACCGCCGAAGAGATTCGGTATGTAGCTTCTGAACTTGAAGCTACTTTAggcggcggtctt                                                                                                                                                                                    >  1:434900/1‑294 (MQ=255)`  
                                                                                                                        `tCCATGATTAGCTCTAAGGTTATCGGCTTAGTTAATCCTGCTGGTATCACCCAGCCACGCCGACTGACCAAAGCTCATACTGGTGACTTCGTTACTGGTCGTCCAGAAGACATCTCGTTCCTCCAACTGGAGAAGCAAGCAGACTTTACTGTAGCTAACGCCGTAAGTGACGCTATCGCGGCTCGCCTTTCGTTTGCCTTTATGTTGAACTCTGCGGTTCAGCGTACAGGTGAACGTGTGACCGCCGAAGAGATTCGGTATGTAGCTTCTGAACTTGAAGATACTTTAGGTGG‑TGTCTAct                                                                                                                                                                                  <  1:721665/301‑1 (MQ=255)`  
                                                                                                                         `ccATGATTAGCTCTAAGGTTATCGGCTTAGTGAATCCTGCTGGTATCACCCAGCCACGCCGACTGACCAAAGCTCAGACTGGTGACTTCGTTACTGGTCGTCCAGAAGACATCTCGTTCCTCCAACTGGAGAAGCAAGCAGACTTTACTGTAGCTAACGCCGTAAGTGACGCTATCGCGGCTCGCCTTTCGTTTGCCTTTATGTTTAACTCTGCGGTTCAGCGTACAGGTGAACGTGTGACCGCCGAAGAGATTCGGTATGTAGCTTCTGAACTTGAAGATACTTTAGGTGG‑TGTCTActc                                                                                                                                                                                 >  1:314904/1‑301 (MQ=255)`  
                                                                                                                         `ccATGATTAGCTCTAAGGTTATCGGCTTAGTGAATCCTGCTGGTATCACCCAGCCACGCCGACTGACCAAAGCTCAGACTGGTGACTTCGTTACTGGTCGTCCAGAAGACATCTCGTTCCTCCAACTGGAGAAGCAAGCAGACTTTACTGTAGCTAACGCCGTAAGTGACGCTATCGAGGCTCGCCTTTCGTTTGCCTTTATGTTGAACTCTGCGGTTCAGCGTACAGGTGAACGTGTGACCGCCGAAGAGATTCGGTATGTAGCTTCTGAACTTGAAGATACTTTAGGTGG‑TGTCTActc                                                                                                                                                                                 <  1:688407/301‑1 (MQ=255)`  
                                                                                                                          `cggagatgtgtataagagacagggCTTAGTGAATCCTGCTGGTATCACCCAGCCACGCCGACTGACCAAAGCTCAGACTGGTGACTTCGTTACTGGTCGTCCAGAAGACATCTCGTTCCTCCAACTGGAGAAGCAAGCAGACTTTACTGTAGCTAACGCCGTAAGTGACGCTATCGCGGCTCGCCTTTCGTTTGCCTTTATGTTGAACTCTGCGGTTCAGCGTACAGGTGAACGTGTGACCGCCGAAGAGATTCGGTATGTAGCTTCTGAACTTGAAGATACTTTAGGTGG‑TGTCTActct                                                                                                                                                                                <  1:108124/279‑1 (MQ=255)`  
                                                                                                                             `gATTAGCTCTAAGGTTATCGGCTTAGTGAATCCTGCTGGTATCACCCAGCCACGCCGACTGACCAAAGCTCAGACTGGTGACTTCGTTACTGGTCGTCCAGAAGACATCTCGTTCCTCCAACTGGAGAAGCAAGCAGACTTTACTGTAGCTAACGCCGTAAGTGACGCTATCGCGGCTCGCCTTTCGTTTGCCTTTATGTTGAACTCTGCGGTTCAGCGTACAGGTGAACGTGTGACCGCCGAAGAGATTCGGTATGTAGCTTCTGAACTTGAAGATACTTTAGGTGG‑TGTCTACTCTATc                                                                                                                                                                             >  1:823974/1‑301 (MQ=255)`  
                                                                                                                              `aTTAGCTCTAAGGTTATCGGCTTAGTGAATCCTGCTGGTATCACCCAGCCACGCCGACTGACCAAAGCTCAGACTGGTGACTTCGTTACTGGTCGTCCAGAAGACATCTCGTTCCTCCAACTGGAGAAGCAAGCAGACTTTACTGTAGCTAACGCCGTAAGTGCCGCTATCGAGGCTCGCCTTTCGTTTGCCTTTATGTTGAACTCTGCGGTTCAGCGTACAGGTGAACGTGTGACCGCCGAAGAGATTCGGTATGTAGCTTCTGAACTTGAAGATACTTTAGGTGG‑TGTCTACTCTATcc                                                                                                                                                                            <  1:249871/301‑1 (MQ=255)`  
                                                                                                                              `aTTAGCTCTAAGGTTATCGGCTTAGTGAATCCTGCTGGTATCACCCAGCCACGCCGACTGACCAAAGCTCAGACTGGTGACTTCGTTACTGGTCGTCCAGAAGACATCTCGTTCCTCCAACTGGAGAAGCAAGCAGACTTTACTGTAGCTAACGCCGTAAGTGACGCTATCGCGGCTCGCCTTTCGTTTGCCTTTATGTTGAACTCTGCGGTTCAGCGTACAGGTGAACGTGTGACCGCCGAAGAGATTCTGTATGTAGCTTCTGAACTTGAAGATACTTTAGGTGG‑TGTCTACTCTATcc                                                                                                                                                                            <  1:123070/301‑1 (MQ=255)`  
                                                                                                                              `aTTAGCTCTAAGGTTATCGGCTTAGTGAATCCTGCTGGTATCACCCAGCCACGCCGACTGACCAAAGCTCAGACTGGTGACTTCGTTACTGGTCGTCCAGAAGACATCTCGTTCCTCCAACTGGAGAAGCAAGCAGACTTTACTGTAGCTAACGCCGTAAGTGACGCTATCGCGGCTCGCCTTTCGTTTGCCTTTATGTTGAACTCTGCGGTTCAGCGTACAGGTGAACGTGTGACCGCCGAAGAGATTCGGTATGTAGCTTCTGAACTTGAAGATACTTTAGGTGG‑TGTCTACTCTATcc                                                                                                                                                                            <  1:816214/301‑1 (MQ=255)`  
                                                                                                                              `aTTAGCTCTAAGGTTATCGGCTTAGTGAATCCTGCTGGTATCACCCAGCCACGCCGACTGACCAAAGCTCAGACTGGTGACTTCGTTACTGGTCGTCCAGAAGACATCTCGTTCCTCCAACTGGAGAAGCAAGCAGACTTTACTGTAGCTAACGCCGTAAGTGACGCTATCGCGGCTCGCCTTTCGTTTGCCTTTATGTTGAACTCTGCGGTTCAGCGTACAGGTGAACGTGTGACCGCCGAAGAGATTCGGTATGTAGCTTCTGAACTTGAAGATACTTTAGGTGG‑TGTCTACTCTATcc                                                                                                                                                                            <  1:116431/301‑1 (MQ=255)`  
                                                                                                                                  `gCTCTAAGGTTATCGGCTTAGTGAATCCTGCTGGTATCACCCAGCCACGCCGACTGACCAAAGCTCAGACTGGTGACTTCGTTACTGGTCGTCCAGAAGACATCTCGTTCCTCCAACTGGAGAAGCAAGCAGACTTTACTGTAGCTAACGCCGTAAGTGACGCTATCGCGGCTCTCCTTTCGTTTGCCTTTATGTTGAACTCTTCGGTTCAGCGTACAGGTGAACGTGTGACCGGCGAAGAGATTCGGTATGTAGCTTCTGAACTTGAAGATACTTTAGGTGG‑TGTCTACTCTATCCTTtc                                                                                                                                                                        >  1:354511/1‑301 (MQ=255)`  
                                                                                                                                  `gCTCTAAGGTTATCGGCTTAGTGAATCCTGCTGGTATCACCCAGCCACGCCGACTGACCAAAGCTCAGACTGGTGACTTCGTTACTGGTCGTCCAGAAGACATCTCGTTCCTCCAACTGGAGAAGCAAGCAGACTTTACTGTAGCTAACGCCGTAAGTGACGCTATCGCGGCTCGCCTTTCGTTTGCCTTTATGTTGAACTCTGCGGTTCAGCGTACAGGTGAACGTGTGACCGCCGAAGAGATTCGGTATGTAGCTTCTGAACTTGAAGATACTTTAGGTGG‑TGTCTACTCTATCCtttt                                                                                                                                                                        >  1:637200/1‑300 (MQ=255)`  
                                                                                                                                  `gCTCTAAGGTTATCGGCTTAGTGAATCCTGCTGGTATCACCCAGCCACGCCGACTGACCAAAGCTCAGACTGGTGACTTCGTTACTGGTCGTCCAGAAGACATCTCGTTCCTCCAACTGGAGAAGCAAGCAGACTTTACTGTAGCTAACGCCGTAAGTGACGCTATCGCGGCTCGCCTTTCGTTTGCCTTTATGTTGAACTCTGCGGTTCAGCGTACAGGTGAACGTGTGACCGCCGAAGAGATTCGGTATGTAGCTTCTGAACTTGAAGATACTTTAGGTGG‑TGTCTACTCTATCCTTtc                                                                                                                                                                        >  1:752735/1‑301 (MQ=255)`  
                                                                                                                                  `gCTCTAAGGTTATCGGCTAAGTGAATCCTGCTGGTATCACCCAGCCACGCCGACTGACCAAAGCTCAGACTGGTGACTTCGTTACTGGTCGTCCAGAAGACATCTCGTTCCTCCAACTGGAGAAGCAAGCAGACTTTACTGTAGCTAACGCCGTAAGTGACGCTATCGCGGCTCGCCTTTCGTTTGCCTTTATGTTGAACTCTGCGGTTCAGCGTACAGGTGAACGTGTGACCGCCGAAGAGATTCGGTATGTAGCTTCTGAACTTGAAGATACTTTAGGTGGCTGTCtcttatacacatc                                                                                                                                                                         >  1:774741/1‑289 (MQ=255)`  
                                                                                                                                       `gggctcggagatgtgtataagagacagggTATCACCCAGCCACGCCGACTGACCAAAGCTCAGACTGGTGACTTCGTTACTGGTCGTCCAGAAGACATCTCGTTCCTCCAACTGGAGAAGCAAGCAGACTTTACTGTAGCTAACGCCGTAAGTGACGCTATCGCGGCTCGCCTTTCGTTTGCCTTTATGTTGAACTCTGCGGTTCAGCGTACAGGTGAACGTGTGACCGCCGAAGAGATTCGGTATGTAGCTTCTGAACTTGAAGATACTTTAGGTGG‑TGTCTACTCTATCCTTTCTCAAg                                                                                                                                                                   <  1:240853/274‑1 (MQ=255)`  
                                                                                                                                          `gTTATCGGCTTAGTGAATCCTGCTGGTATCACCCAGCCACGCCGACTGACCAAAGCTCAGACTGGTGACTTCGTTACTGGTCGTCCAGAAGACATCTCGTTCCTCCAACTGGAGAAGCAAGCAGACTTTACTGTAGCTAACGCCGTAAGTGACGCTATCGCGGCTCGCCTTTCGTTTGCCTTTATGTTGAACTCTGCGGTTCAGCGTACAGGTGAACGTGTGACCGCCGAAGAGATTCGGTATGTAGCTTCTGTACTTGACGCTACTTTAGGTGG‑TGTCTACTCTATCCTTTCTCAAGAAt                                                                                                                                                                >  1:28585/1‑301 (MQ=255)`  
                                                                                                                                                `ggtgtgtataagagacagggTATCACCCACCCACGCCGACTGACCAAAGCTCAGACTGGTGACTTCGTTACTGGGCGTCCAGAAGACATCTCGTTCCTCCAACTGGAGAAGCAAGCAGACTTTACTGTAGCTAACGCCGTAAGTGACGCTATCGCGGCTCGCCTTTCGTTTGCCTTTATGTTGAACTCTGCGGTTCAGCGTACAGGTGAACGTGTGACCGCCGAAGAGATTCGGTATGTAGCTTCTGAACTTGAAGATACTTTAGGTGG‑TGTCTACTCTATCCTTTCTCAAGAATTACAAt                                                                                                                                                          <  1:679619/283‑1 (MQ=255)`  
                                                                                                                                                  `cTTAGTGAATCCTGCTGGTATCACCCAGCCACGCCGACTGACCAAAGCTCAGACTGGTGACTTCGTTACTGGTCGTCCAGAAGACATCTCGTTCCTCCAACTGGAGAAGCAAGCAGACTTTACTGTAGCTAACGCCGTAAGTGAAGCTATCGCGGATAGCCTTTCGTTTGCCTTTATGTTGAACTCTGCGGTTCAGCGTACAGGTGAACGTGTGACCGCCGAAGAGATTCGGTATGTAGCTTCTGAACTTGAAGATACTTTAGGTGG‑TGTCTACTCTATCCTTTCTCAAGAATTACAATTg                                                                                                                                                        <  1:745976/301‑1 (MQ=255)`  
                                                                                                                                                         `aaTCCTGCTGGTATCACCCAGCCACGCCGACTGACCAAAGCTCAGACTGGTGACTTCGTTACTGGTCGTACAGAAGACATCTCGTTCCTCCAACTGGAGAAGCAAGCAGACTTTACTGTAGCTAACGCAGTAAGTGACGCTATCGCGGCTCGCCTTTCGTTTGCCTTTATGTTGAACTCTGCGGTTCAGCGTACAGGTGAACGTGTGACCGCCGAAGAGATTCGGTATGTAGCTTCTGAACTTGAAGATACTTTAGGTGG‑TGTCTACTCTATCCTTTCTCAAGAATTACAATTGCCTCTgg                                                                                                                                                 <  1:135125/301‑1 (MQ=255)`  
                                                                                                                                                           `tCCTGCTGGTATCACCCAGCCACGCCGACTGACCAAAGCTCAGACTGGTGACTTCGTTACTGGTCGTCCAGAAGACATCTCGTTCCTCCAACTGGAGAAGCAAGCAGACTTTACTGTAGCTAACGCCGTAAGTGACGCTATCGCGGCTCGCCTTTCGTTTGCCTTTATGTTGAACTCTGCGGTTCAGCGTACAGGTGAACGTGTGACCGCCGAAGAGATTCGGTATGTAGCTTCTGAACTTGAAGATACTTTAGGTGG‑TGTCTACTCTATCCTTTCTCAAGAATTACAATTGCCTCTGGTa                                                                                                                                               >  1:323255/1‑301 (MQ=255)`  
                                                                                                                                                           `aaagagacagaTCACCCAGCCACGCCGACTGACCAAAGCTCAGACTGGTGACTTCGTTACTGGTCGTCCAGAAGACATCTCGTTCCTCCAACTGGAGAAGCAAGCAGACTTTACTGTAGCTAACGCCGTAAGTGACGCTATCGCGGCTCGCCTTTCGTTTGCCTTTATGTTGAACTCTGCGGTTCAGCGTACAGGTGAACGTGTGACCGCCGAAGAGATTCGGTATGTAGCTTCTGAACTTGAAGATACTTTAGGTGG‑TGTCTACTCTATCCTTTCTCAAGAATTACAATTGCCTCTGGTa                                                                                                                                               <  1:730386/291‑1 (MQ=255)`  
                                                                                                                                                            `ccTGCTGGTATCACCCAGCCACGCCGACTGACCAAAGCTCAGACTGGTGACTTCGTTACTGGTCGTCCAGAAGACATCTCGTTCCTCCAACTGGAGAAGCAAGCAGACTTTACTGTAGCTAACGCCGTAAGTGACGCTATCGCGGCTCGCCTTTCGTTTGCCTTTATGTTGAACTCTGCGGTTCAGCGTACAGGTGAACGTGTGACCGCCGAAGAGATTCGGTATGTAGCTTCTGAACTTGAAGATACTTTAGGTGG‑TGTCTACTCTATCCTTTCTCAAGAATTACAATTGCCTCTGGTAc                                                                                                                                              <  1:307275/301‑1 (MQ=255)`  
                                                                                                                                                              `tgctgGTATCACCCAGCCACGCCGACTGACCAAAGCTCAGACTGGTGACTTCGTTACTGGTCGTCCAGAAGACATCTCGTTCCTCCAACTGGAGAAGCAAGCAGACTTTACTGTAGCTAACGCCGTAAGTGACGCTATCGCGGCTCGCCTTTCGTTTGCCTTTATGTTGAACTCTGCGGTTCAGCGTACAGGTGAACGTGTGACCGCCGAAGAGATTCGGTATGTAGCTTCTGAACTTGAAGATACTTTAGGTGG‑TGTCTACTCTATCCTTTCTCAAGAATTACAATTGCCTCTGTTActa                                                                                                                                            >  1:330983/1‑299 (MQ=255)`  
                                                                                                                                                               `gctgGTATCACCCAGCCACGCCGACTGACCAAAGCTCAGACTGGTGACTTAGTTACTGGTCGTCCAGAAGACATCTCGTTCCTGCAACTGGAGAAGCAAGCAGACTTTACTGTAGCTAACGCCGTAAGTGACGCTATCGCGGCTCGCCTTTCGTTTGCCTTTATGTTGAACTCTTCGGTTCAGCGTACAGGTGAACGTGTGACCTCCGAAGAGATTCGGTATGTAGCTTCTGAACTTGAAGATACTTTAGGTGG‑TGTCTACTCTATCCTTTCTCAAGAATTACAATTGCCTCTGGTACGAg                                                                                                                                           <  1:646834/301‑1 (MQ=255)`  
                                                                                                                                                                       `cACCCAGCCACGCCGACTGACCAAAGCTCAGACTGGTGACTTCGTTACTGGTCGTCCAGAAGACATCTCGTTCCTCCAACTGGAGAAGCAAGCAGACTTTACTGTAGCTAACGCCGTAAGTGACGCTATCGCGGCTCGCCTTTCGTTTGCCTTTATGTTGAACTCTGCGGTTCAGCGTACAGGTGAACGTGTGACCGCCGAAGAGATTCGGTATGTAGCTTCTGAACTTGAAGATACTTTAGGTGG‑TGTCTACTCTATCCTTTCTCAAGAATTACAATTGCCTCTGGTACGAgctgtctct                                                                                                                                   >  1:171338/1‑293 (MQ=255)`  
                                                                                                                                                                          `ataagagacaggACTGACCAAAGCTCAGACTGGTGACTTCGTTACTGGTCTTCCAGAAGACATCTCGTTCCTCCAACTGGAGAAGCAAGCAGACTTTACTGTAGCTAACGCCGTAAGTGACGCTATCGCGGCTCGCCTTTCGTTTGCCTTTATGTTGAACTCTGCGGTTCAGCGTACAGGTGAACGTGTGACCGCCTAAGAGATTCGGTATGTAGCTTCTGAACTTGAAGATACTTTAGGTGG‑TGTCTACTCTATCCTTTCTCAAGAATTACAATTGCCTCTGGTACGAGTGCTCTTGAAg                                                                                                                                <  1:55331/290‑1 (MQ=255)`  
                                                                                                                                                                                      `aCTGACCAAAGCTCAGACTGGTGACTTCGTTACTGGTCGTCCAGAAGACATCTCGTTCCTCCAACTGGAGAAGCAAGCAGACTTTACTGTAGCTAACGCCGTAAGTGACGCTATCGCGGCTCGCCTTTCGTTTGCCTTTATGTTGAACTCTGCGGTTCAGCGTACAGGTGAACGTGTGACCGCCGAAGAGATTCGGTATGTAGCTTCTGAACTTGAAGATACTTTAGGTGG‑TGTCTACTCTATCCTTTCTCAAGAATTACAATTGCCTCTGGTACGAGTGCTCTTGAAGCAACTACAAGcc                                                                                                                    <  1:265028/301‑1 (MQ=255)`  
                                                                                                                                                                                      `aCTGACCAAAGCTCAGACTGGTGACTTCGTTACTGGTCGTCCAGAAGACATCTCGTTCCTCCAACTGGAGAAGCAAGCAGACTTAACTGTAGCTAACGCCGTAAGTGACGCTATCGCGGCTCGCCTTTCGTTTGCCTTTATGTTGAACTCTGCGGTTCAGCGTACAGGTGAACGTGTGACCGCCGAAGAGATTCGGTATGTAGCTTCTGAACTTGAAGATACTTTAGGTGG‑TGTCTACTCTATCCTTTCTCAAGAATTACAATTGCCTCTGGTACGAGTGCTCTTGAAGCAACTACAAGcc                                                                                                                    <  1:216250/301‑1 (MQ=255)`  
                                                                                                                                                                                               `aGCTCGGACTGGTGACTTCGTTACTGGTCGTCCAGAAGACATCTCGTTCCTCCAACTGGAGAAGCAAGCAGACTTTACTGTAGCTAACGCCGTAAGTGACGCTATCGCGGCTCGCCTTTCGTTTGCCTTTATGTTGAACTCTGCGGTTCAGCGTACAGGTGAACGTGTGACCGCCGAAGAGATTCGGTATGTAGCTTCTGAACTTGAAGATACTTTAGGTGG‑TGTCTACTCTATCCTTTCTCAAGAATTACAATTGCCTCTGGTACGAGTGCTCTTGAAGCAACTACAAGCCACGCAAAAg                                                                                                           >  1:193336/1‑301 (MQ=255)`  
                                                                                                                                                                                               `aGCTCAGACTGGTGACTTCGTTACTGGTCGTCCAGAAGACATCTCGTTCCTCCAACTGGAGAAGCAAGCAGACTTTACTGTAGCTAACGCCGTAAGTGACGCTATCGCGGCTCGCCTTTCGTTTGCCTTTATGTTGAACTCTGCGGTTCAGCGTACAGGTGAACGTGTGACCGCCGAAGAGATTCGGTATGTAGCTTCTGAACTTGAAGATACTTTAGGTGG‑TTTCTACTCTATCCTTTCTCAAGAATTACAATTGCCTCTGGTCCGAGTGCTCTTGAAGCCACTACTACCCACGCAACat                                                                                                           >  1:62953/1‑300 (MQ=255)`  
                                                                                                                                                                                                `gCTCAGACTGGTGACTTCGTTACTGGTCGTCCAGAAGACATCTCGTTCCTCCAACTGGAGAAGCAAGCAGACTTTACTGTAGCTAACGCCGTAAGTGACGCTATCGCGGCTCGCGTTTCGTTTGCCTTTATGTTGAACTCTGCGGTTCAGCGTACAGGTGAACGTGTGACAGCCGAAGAGATTCGGTATGTAGCTTCTGAACTTGAAGATACTTTAGGTGG‑TGTCTACTCTATCCTTTCTCAAGAATTACAATTGCCTCTGGTACGCGTGCTCTTGAAGCAACTACAAGCCACGCAAAAGa                                                                                                          >  1:521410/1‑301 (MQ=255)`  
                                                                                                                                                                                                   `cAGACTGGTGACTTCGTTACTGGTCGTCCAGAAGACATCTCGTTCCTCCAACTGGAGAAGCAAGCAGACTTTACTGTAGCTAACGCCGTAAGTGACGCTATCGCGGCTCGCCTTTCGTTTGCCTTTATGTTGAACTCTGCGGTTCAGCGTACAGGTGAACGTGTGACCGCCGAAGAGATTCGGTATGTAGCTTCTGAACTTGAAGATACTTTAGGTGG‑TGTCTACTCTATCCTTTCTCAAGAATTACAATTGCCTCTGGTACGAGTGCTCTTGAAGCAACTACAAGCCACGCAACAGATTc                                                                                                       <  1:375588/301‑1 (MQ=255)`  
                                                                                                                                                                                                         `ggTGACTTCGTTACTGGTCGTCCAGAAGACATCTCGTTCCTCCAACTGGAGAAGCAAGCAGACTTTACTGTAGCTAACGCCGTAAGTGACGCTATCGCGGCTCGCCTTTCGTTTGCCTTTATGTTGAACTCTGCGGTTCAGCGTACAGGTGAACGTGTGACCGCCGAAGAGATTCGGTATGTAGCTTCTGAACTTGAAGATACTTTAGGTGG‑TGTCTACTCTATCCTTTCTCAAGAATTACAATTGCCTCTGGTACGAGTGCTCTTGAAGCAACTACAAGCCACGCAACAGATTctgtctc                                                                                                 >  1:27272/1‑295 (MQ=255)`  
                                                                                                                                                                                                              `ggtataagagacaggtcCAGAAGACATCTCGTTCCTCCAACTGGAGAAGCAAGCAGACTTTACTGTAGCTAACGCCGTAAGTGACGCTATCGCGGCTCGCCTTTCGTTTGCCTTTATGTTGAACTCTGCGGTTCAGCGTACAGGTGAACGTGTGACCGCCGAAGAGATTCGGTATGTAGCTTCTGAACTTGAAGATACTTTAGGTGG‑TGTCTACTCTATCCTTTCTCAAGAATTACAATTGCCTCTGGTACGAGTGCTCTTGAAGCAACTACAAGCCACGCAACAGATTCCTGAGTTACCt                                                                                            <  1:599326/287‑1 (MQ=255)`  
                                                                                                                                                                                                              `cTTCGTTACTGGTCGTCAAGAAGACATCTCGTTCCTCCAACTGGAGAAGCAAGCAGACTTTACTGTAGCTAACGCCGTAAGTGACGCTATCGCGGCTCGCCTTTCGTTTGCCTTTATGTTGAACTCTGCGGTTCAGCGTACAGGTGAACGTGTGACCGCCGAAGAGATTCGGTATGTAGCTTCTGAACTTGAAGATACTTTAGGTGG‑TGTCTACTCTATCCTTTCTCAAGAATTACAATTGCCTCTGGTACGAGTGCTCTTGAAGCAACTACAAGCCACGCAACAGATTCCTGAGTTACCt                                                                                            >  1:150097/1‑301 (MQ=255)`  
                                                                                                                                                                                                                 `cGTTACTGGTCGTCCAGAAGACATCTCGTTCCTCCAACTGGAGAAGCAAGCAGACTTTACTGTAGCTAACGCCGTAAGTGACGCTATCGCGGCTCGCCTTTCGTTTGCCTTTATGTTGAACTCTGCGGTTCAGCGTACAGGTGAACGTGTGACCGCCGAAGAGATTCGGTATGTAGCTTCTGAACTTGAAGATACTTTAGGTGG‑TGTCTACTCTATCCTTTCTCAAGAATTACAATTGCCTCTGGTACGAGTGCTCTTGAAGCAACTACAAGCCACGCAACAGATTCCTGAGTTACCTAAg                                                                                         >  1:738900/1‑301 (MQ=255)`  
                                                                                                                                                                                                                   `ttACTGGTCGTCCAGAAGACATCTCGTTCCTCCAACTGGAGAAGCAAGCAGACTTTACTGTAGCTAACGCCGTAAGTGACGCTATCGCGGCTCGCCTTTCGTTTGCCTTTATGTTGAACTCTGCGGTTCAGCGTACAGGTGAACGTGTGACCGCCGAAGAGATTCGGTATGTAGCTTCTGAACTTGAAGATACTTTAGGTGG‑TGTCTACTCTATCCTTTCTCAAGAATTACAATTGCCTCTGGTACGAGTGCTCTTGAAGCAACTACAAGCCACGCAACAGATTCCTGAGTTACCTAAGGa                                                                                       <  1:225819/301‑1 (MQ=255)`  
                                                                                                                                                                                                                   `ttACTGGTCGTCCAGAAGACATCTCGTTCCTACAACTGGAGAAGCAAGCAGACTTTACTGTAGCTAACGCCGTAAGTGACGCTATCGCGGCTCGCCTTTCGTTTGCCTTTATATTGAACTCTGCGGTTCAGCGTACAGGTGAACGTGTGACCGCCGAAGAGATTCGGTATGTAGCTTCTGAACTTGAAGATACTTTAGGTGG‑TGTCTACTCTATCCTTTCTCAAGAATTACAATTGCCTCTGGTACGAGTGCTCTTGAAGCAACTACAAGCCACGCAACAGATTCCTGAGTTACCTAAGGa                                                                                       <  1:279890/301‑1 (MQ=255)`  
                                                                                                                                                                                                                            `gtcCAGAAGACATCTCGTTCCTCCAACTGGAGAAGCAAGCAGACTTTACTGTAGCTAACGCCGTAAGTGCCGCTATCGAGGCTCGCCTTTCGTTTGCCTTTATGTTGAACTCTGAGGTTCAGCGTATAGGTGAACGTGTGAGCGCCGAAGAGATTCGGTATGTAGATTCTGAACTTGAAGATACTTTAGGTGG‑TGTCTACTCTATCCTTTCTCAAGAATTACAATTGCCTCTGGTACGAGTGCTCTTGAAGCAACTACAAGCCACGCAACAGATTCATGAGTTACATAAGGAAGCCGTaga                                                                              >  1:544698/1‑301 (MQ=255)`  
                                                                                                                                                                                                                            `gtcCAGAAGACATCTCGTTCCTCCAACTGGAGAAGCAAGCAGACTTTACTGTAGCTAACGCCGTAAGTGACGCTATCGTGGCTCGCCTTTCGTTTGCCTTTATGTTGAACTCTGCGGTTCAGCGTACAGGTGAACGTGTGACCGCCGAAGAGATTCGGTATGTAGCTTCTGAACTTGAAGATACTTTAGGTGG‑TGTCTACTCTATCCTTTCTCAAGAATTACAATTTCCTCTGGTACGAGTGCTCTTGAAGCAACTACAAGCCACGCAACAGATTCCTGAGTTACCTAAGGAAGCCGTaga                                                                              >  1:670522/1‑301 (MQ=255)`  
                                                                                                                                                                                                                              `cgaaaggaCGTCTCGTTCGTCCAACTGGTGAAGCAAGCCGACTTTAATGAAGCTAACGCCGTAAGTGGCGCTATCGCAGCTCGCATTTCGTTTGCCTTTATGTTGAACTCTGCGGTTCAGCGTACAAGTGAATGTGTTACCGACGAAGATGGTCGTTATGAAGCTTCTGAACTTGAAGATACTTTAGGTGG‑TGTCTACTCTATGCTTTCTCAAGAATTACGATTGCCTCTGGTACGAGTGCTCTTGAAGCAACTACAAGCCACGCAACAGATTCCTGAGTTACCTAAGGAAGCCGTAGAGc                                                                            <  1:458196/295‑1 (MQ=255)`  
                                                                                                                                                                                                                              `ccAGAAGACATCTCGTTCCTCCAACTGGAGAAGCAAGCAGACTTTACTGTAGCTAACGCCGTAAGTGCCGCTATCGAGGCTCGCCTTTCGTTTGCCTTTATGTTGAACTCTGCGGTTCAGCGTACAGGTGAACGTGTGACCGCCGAAGAGATTCGGTATGTAGCTTCTGAACTTGAAGATACTTTAGGTGG‑TGTCTACTCTATCCTTTCTCAAGAATTACAATTGCCTCTGGTACGAGTGCTCTTGAAGCAACTACAAGCCACGCAACAGATTCCTGAGTTACCTAAGGAAGCCGTAGAGc                                                                            <  1:801026/301‑1 (MQ=255)`  
                                                                                                                                                                                                                              `ccAGAAGACATCTCGTTCCTCCAACTGGAGAAGCAAGCAGACTTTACTGTAGCTAACGCCGTAAGTGACGCTATCGCGGCTCGCCTTTCGTTTGCCTTTATGTTGAACTCTGCGGTTCAGCGTACAGGTGAACGTGTGACCGCCGAAGAGATTCGGTATGTAGTTTCTGAACTTGAAGATACTTTAGGTGG‑TGTCTACTCTATCCTTTCTCAAGAATTACAATTGCCTCTGGTACGAGTGCTCTTGAAGCAACTACAAGCCACGCAACAGATTCCTGAGTTACCTAAGGAAGCCGTAGAGc                                                                            <  1:338544/301‑1 (MQ=255)`  
                                                                                                                                                                                                                              `ccAGAAGACATCTCGTTCCTCCAACTGGAGAAGCAAGCAGACTTTACTGTAGCTAACGCCGTAAGGGACGCTATCGCGGCTCGCCTTTCGTTTTTCTTTATGTTGGACTCTGCGGTTCAGCGGTCAGGTGAACGTGTGACCGCCGGAGTGATTCGGTATGTAGCTTCTGAACTTGAAGATACTTTAGGTGG‑TGTCTACTCTATCCTTTCTCAAGAATTACAATTGCCTCTGGTACGAGTGCTCTTGAAGCAACTACAAGCCACGCAACAGATTCCTGAGTTACCTAAGGAAGCCGTAGAGc                                                                            <  1:56641/301‑1 (MQ=255)`  
                                                                                                                                                                                                                                 `gaagaCATCTCGTTCCTCCAACTGGAGAAGCAAGCAGACTTTACTGTAGCTAACGCCGTAAGTTACGCTATCGCGGCTCGCCTTTCGTTTGCCTTTATGTTGAACTCTGCGGTTCAGCGTACAGGTGAACGTGTGACCGCCGAAGAGATTCGGTATGTAGCTTCTGAACTTGAAGATACTTTAGGTGG‑TGTCTACTCTATCCTTTCTCAAGCATTACAATTGCCTCTGGTACGAGTGCTCTTGAAGCAACTACAAGCCACGCAACAGATTCCTGAGTTACCTAAGGaactttctcttatac                                                                         >  1:291667/1‑288 (MQ=255)`  
                                                                                                                                                                                                                                 `gaagaCATCTCGTTCCTCCAACTGGAGAAGCAAGCAGACTTTACTGTAGCTAACGCCGTAAGTGACGCTATCGCGGCTCGCCTTTCGTTTGCCTTTATGTTGAACTCTGCGGTTCAGCGTACAGGTGAACGTGTGACCGCCGAAGAGATTCGGTATGTAGCTTCTGAACTTGAAGATACTTTAGGTGG‑TGTCTACTCTATCCTTTCTCAAGAATTACAATTGCCTCTGGTATGAGTGCTCTTGAAGCAACTACAAGCCACGCAACAGAATCCTGAGTTACCTAAGGAAGCCGTAGAGCCaa                                                                         >  1:477990/1‑301 (MQ=255)`  
                                                                                                                                                                                                                                              `tcctccAACTGGAGAAGCAAGCAGACTTTACTGTAGCTAACGCCGTAAGTGACGCTATCGCGGCTCGCCTTTCGTTTGCCTTTATGTTGAACTCTGCGGTTCAGCGTACAGGTGAACGTGTGACCGCCGAAGAGATTCGGTATGTAGCTTCTGAACTTGAAGATACTTTAGGTGG‑TGTCTACTCTATCCTTTCTCAAGAATTACAATTGCCTCTGGTACGAGTGCTCTTGAAGCAACTACAAGCCACGCAACAGATTCCTGAGTTACCTAAGGAAGCCGTAGAGCCAACCATTAGTACAgg                                                            <  1:538831/301‑1 (MQ=255)`  
                                                                                                                                                                                                                                              `tcctccAACTGGAGAAGCAAGCAGACTTTACTGTAGCTAACGCCGTAAGTGACGCTATCGCGGCTCGCCTTTCGTTTGCCTTTATGTTGAACTCTGCGGTTCAGCGTACAGGTGAACGTGTGACCGCCGAAGAGATTCGGTATGTAGCTTCTGAACTTGAAGATACTTTAGGTGG‑TGTCTACTCTATCCTTTCTCAAGAATTACAATTGCCTCTGGTACGAGTGCTCTTGAAGCAACTACAAGCCACGCAACAGATTCCTGAGTTACCTAAGGAAGCCGTAGAGCCAACCATTAGTACAgg                                                            >  1:208465/1‑301 (MQ=255)`  
                                                                                                                                                                                                                                                 `tccAACTGGAGAAGCAAGCAGACTTTACTGTAGCTAACGCCGTAAGTGACGCTATCGCGGCTCGCCTTTCGTTTGCCTTTATGTTGAACTCTGCGGTTCAGCGTACAGGTGAACGTGTGACCGCCGAAGAGATTCGGTATGTAGCTTCTGAACTTGAAGATACTTTAGGTGG‑TGTCTACTCTATCCTTTCTCAAGAATTACAATTGCCTCTGTTACGAGTGCTCTTGAAGCAACTACAAGCCACGCAACAGATTCCTGAGTTACCTAAGGAAGCCGTAGAGCCAACCATTAGTACAGGTCt                                                         >  1:768552/1‑301 (MQ=255)`  
                                                                                                                                                                                                                                                   `cAACTGGAGAAGCAAGCAGACTTTACTGTAGCTAACGCCGTAAGTGACGCTATCGCGGCTCGCCTTTCGTTTGCCTTTATGTTGAACTCTGCGGTTCAGCGTACAGGTGAACGTGTGACCGCCGAAGAGATTCGGTATGTAGCTTCTGAACTTGAAGATACTTTAGGTGG‑TGTCTACTCTATCCTTTCTCAAGAATTACAATTGCCTCTGGTACGAGTGCTCTTGAAGCAACTACAAGCCACGCAACAGATTCCTGAGTTACCTAAGGAAGCCGTAGAGCCAACCATTAGTACAGGTCTgg                                                       >  1:141839/1‑301 (MQ=255)`  
                                                                                                                                                                                                                                                      `cTGGAGAAGCAAGCAGACTTTACTGTAGCTAACGCCGTAAGTGACGCTATCGCGGCTCGCCTTTCGTTTGCCTTTATGTTGAACTCTGCGGTTCAGCGTACAGGTGAACGTGTGACCGCCGAAGAGATTCGGTATGTAGCTTCTGAACTTGAAGATACTTTAGGTGG‑TGTCTACTCTATCCTTTCTCAAGAATTACAATTGCCTCTGGTACGAGTGCTCTTGAAGCAACTACAAGCCACGCAACAGATTCCTGAGTTACCTAAGGAAGCCGTAGAGCCAACCATTAGTACAGGTCTGGAAg                                                    >  1:788757/1‑301 (MQ=255)`  
                                                                                                                                                                                                                                                           `gaAGCAAGCAGACTTTACTGTAGCTAACGCCGTAAGTGACGCTATCGCGGCTCGCCTTTCGTTTGCCTTTATGTTGAACTCTGCGGTTCAGCGTACAGGTGAACGTGTGACCGCCGAAGAGATTCGGTATGTAGCTTCTGAACTTGAAGATACTTTAGGTGG‑TGTCTACTCTATCCTTTCTCAAGAATTACAATTGCCTCTGGTACGAGTGCTCTTGAAGCAACTACAAACCACGCAACAGATTCCTGAGTTACCTAAGGAAGCCGTAGAGCCAACCATTAGTACCTGTCtcttatacaca                                               >  1:615034/1‑291 (MQ=255)`  
                                                                                                                                                                                                                                                                    `aGACTTTACTGTAGCTAACGCCGTAAGGGCCGCTATAGAGGCTGGCTTTTCTTTTGCTTTTATGTTGAACTTGGCGGTTCAGCGTAAAGGTGAACGGGTGACCGCCGAAGAGACTCGGTATGTAGCTTCGGAATTTGAAGATACTTTAGGTGG‑TGTTTACTCTATCCTTTCTCAAGAATTACAATTGCCTCTGTTACGAGTGCTCTTGAAGCAACTACAAGCCACGAAACAGATTCCTGAGTTACCT‑AGGAAGCCGTAGAGCCAACCATTAGTACAGGTCTGGAAGCAATTGGTCGAGGAc                                     <  1:49359/301‑1 (MQ=255)`  
                                                                                                                                                                                                                                                                     `gACTTTACTGTAGCTAACGCCGTAAGTGACGCTATCGCGGCTCGCCTTTCGTTTGCCTTTATGTTGAACTCTGCGGTTCAGCGTACAGGTGAACGTGTGACCGCCGAAGAGATTCGGTATGTAGCTTCTGAACTTGAAGATACTTTAGGTGG‑TGTCTACTCTATCCTTTCTCAAGAATTACAATTGCCTCTGGTACGAGTGCTCTTGAAGCAACTACAAGCCACGCAACAGATTACTGAGTTACCTAAGGAAGCCGTAGAGCCAACCATTAGTACAGGTCTGGAAGCAATTGGTCGAGGAc                                     <  1:794738/301‑1 (MQ=255)`  
                                                                                                                                                                                                                                                                       `cTTTACTGTAGCTAACGCCGTAAGTGGCGCTATCGAGGATCGCCTTTCGTTTGCCTTTATGTTGAACTCTGCGGTTCAGCGTACAGGTGAACGTGTGACCGCTGAAGAGATTCGGTATGTAGCTTCTGAACTTGAAGATACTTTAGGTGG‑TGTCTACTCTATCCTTTCTCAAGAATTACAATTGCCTCTGGTACGAGTGCTCTTGAAGCAACTACAAGCCACGCAACAGATTCCTGAGTTACCTAAGGAAGCCGTAGAGCCAACCATTAGTACAGGTCTGGAAGCAATTGGTTGAGGACaa                                   >  1:709692/1‑301 (MQ=255)`  
                                                                                                                                                                                                                                                                              `gTAGCTAACGCCGTAAGTGACGCTATCTCGGCTCGCCTTTCGTTTGCCTTTATGTTGAACTCTGCGGTTCAGCGTACAGGTGAACGTGTGACCGCCGAAGAGATTCGGTATGTAGCTTCTGAACTTGAAGATACTTTAGGTGG‑TGTCTACTCTATCCTTTCTCAAGAATTACAATTGCCTCTGGTACGAGTGCTCTTGAAGCAACTACAAGCCACGCAACAGATTCCTGAGTTACCTAAGGAATCCGTAGAGCCAACCATTAGTACAGGTCTGGAAGCAATTGGTCGAGGACAAGACCTTg                            >  1:621941/1‑301 (MQ=255)`  
                                                                                                                                                                                                                                                                                `aGCTAACGCCGTAAGTGACGCTATCGCGGCTCGCCTTTCGTTTGCCTTTATGTTGAACTCTGCGGTTCAGCGTACAGGTGAACGTGTGACCGCCGAAGAGATTCGGTATGTAGCTTCTGAACTTGAAGATACTTTAGGTGG‑TGTCTACTCTATCCTTTCTCAAGAATTACAATTGCCTCTGGTACGAGTGCTCTTGAAGCAACTACAAGCCACGCAACAGATTCCTGAGTTACCTAAGGAAGCCGTAGAGCCAACCATTAGTACAGGTCTGGAAGCAATTGGTCGAGGACAAGACCTTGAt                          <  1:574142/301‑1 (MQ=255)`  
                                                                                                                                                                                                                                                                                    `aacgCCGTAAGTGACGCTATCGCGGCTCGCCTTTCGTTTGCCTTTATGTTGAACTCTGCGGTTCAGCGTACAGGTGAACGTGTGACCGCCGAAGAGATTCGGTATGTAGCTTCTGAACTTGAAGATACTTTAGGTGG‑TGTCTACTCTATCCTTTCTCAAGAATTACAATTGCCTCTGGTACGAGTGCTCTTGAAGCAACTACAAGCCACGCAACAGATTCCTGAGTTACCTAAGGAAGCCGTAGAGCCAACCATTAGTACAGGTCTGGAAGCAATTGGTCGAGGACAAGACCTTGATAAGc                      <  1:662707/298‑1 (MQ=255)`  
                                                                                                                                                                                                                                                                                      `cgCCGTAAGTGACGCTATCGCGGCTCGCCTTTCGTTTGCCTTTATGTTGAACTCTGCGGTTCAGCGTACAGGTGAACGTGTGACCGCCGAAGAGATTCGGTATGTAGCTTCTGAACTTGAAGATACTTTAGGTGG‑TGTCTACTCTATCCTTTCTCAAGAATTACAATTGCCTCTGGTACGAGTGCTCTTGAAGCAACTACAAGCCACGCAACAGATTCCTGAGTTACCTAAGGAAGCCGTAGAGCCAACCATTAGTACAGGTCTGGAAGCAATTGGTCGAGGACAAGACCTTGATAAGcct                    >  1:761491/2‑299 (MQ=255)`  
                                                                                                                                                                                                                                                                                      `cgCCGTAAGTGACGCTATCGCGGCTCGCCTTTCGTTTGCCTTTATGTTGAACTCTGCGGTTCAGCGTACAGGTGAACGTGTGACCGCCGAAGAGATTCGGTATGTAGCTTCTGAACTTGAAGATACTTTAGGTGG‑TGTCTACTCTATCCTTTCTCAAGAATTACAATTGCCTCTGGTACGAGTGCTCTTGAAGCAACTACAAGCCACGCAACAGATTCCTGAGTTACCTAAGGAAGCCGTAGAGCCAACCATTAGTACAGGTCTGGAAGCAATTGGTCGAGGACAAGACCTTGATAAGCTg                    >  1:300491/2‑301 (MQ=255)`  
                                                                                                                                                                                                                                                                                          `tataagagacagaTCGCGGCTCGCCTTTCGTTTGCCTTTATGTTGAACTCTGCGGTTCAGCGTACAGGTGAACGTGTGACCGCCGAAGAGATTCGGTATGTAGCTTCTGAACTTGAAGATACTTTAGGTGG‑TGTCTACTCTATCCTTTCTCAAGAATTACAATTGCCTCTGGTACGAGTGCTCTTGAAGCAACTACAAGCCACGCAACAGATTCCTGAGTTACCTAAGGAAGCCGTAGAGCCAACCATTAGTACAGGTCTGGAAGCAATTGGTCGAGGACAAGACCTTGATAAGCTGGAGc                <  1:607777/289‑1 (MQ=255)`  
                                                                                                                                                                                                                                                                                            `aattGACGCTATCTCGGCTCGCCTTTCGTTTGCCTTTATGTTGAACTCTGCGGTTCAGCGTACAGGTGAACGTGTGACCGCCGAAGAGATTCGGTATGTAGCTTCTGAACTTGAAGATACTTTAGGTGG‑TGTCTACTCTATCCTTTCTCAAGAATTACAATTGCCTCTGGTACGAGTGCTCTTGAAGCAACTACAAGCCACTCAACAGATTCCTGAGTTACCTAAGGAAGCCGTAGAGCCAACCATTAGTACAGGTCTGGAAGCAATTGGTCGAGTACAAGACCTTGATAAGCTGGAGCgg              <  1:413743/298‑1 (MQ=255)`  
                                                                                                                                                                                                                                                                                              `gTGATGCTATCGCGGCTCGCCTTTCGTTTGCCTTTATGTTGAACTCTGCGGTTCAGCGTACAGGTGAACGTGTGACCGCCGAAGAGATTCGGTATGTAGCTTCTGAACTTGAAGATACTTTAGGTGG‑TGTCTACTCTATCCTTTCTCAAGAATTACAATTGCCTCTGGTACGAGTGCTCTTGAAGCAACTACAAGCCACGCAACAGATTCCTGAGTTACCTAAGGAAGCCGTAGAGCCAACCATTAGTACAGGTCTGGAAGCAATTGGTCGAGGACAAGACCTTGATAAGCTGGAGCGgtg            >  1:442061/1‑301 (MQ=255)`  
                                                                                                                                                                                                                                                                                                    `cTATCGCGGCTCGCCTTTCGTTTGCCTTTATGTTGAACTCTGCGGTTCAGCGTACAGGTGAACGTGTGACCGCCGAAGAGATTCGGTATGTAGCTTCTGAACTTGAAGATACTTTAGGTGG‑TGTCTACTCTATCCTTTCTCAAGAATTACAATTGCCTCTGGTACGAGTGCTCTTGAAGCAACTACAAGCCACGCAACAGATTCCTGAGTTACCTAAGGAAGCCGTAGAGCCAACCATTAGTACAGGTCTGGAAGCAATTGGTCGAGGACAAGACCTTGATAAGCTGGAGCGGTGTGTCAc      <  1:781635/301‑1 (MQ=255)`  

                                                                                                                                                                                                                                                                                                          `|`                                                                                                                                                                                                                                                                                                             
`CCATACATCCCGATTCGGATGGTCAGACTAGATGGTGAATCCTACGGTCGTTCGTACATTGAGGAATACTTAGGTGACTTACGGTCCCTTGAAAATCTCCAAGAGGCTATCGTCAAGATGTCCATGATTAGCTCTAAGGTTATCGGCTTAGTGAATCCTGCTGGTATCACCCAGCCACGCCGACTGACCAAAGCTCAGACTGGTGACTTCGTTACTGGTCGTCCAGAAGACATCTCGTTCCTCCAACTGGAGAAGCAAGCAGACTTTACTGTAGCTAAAGCCGTAAGTGACGCTATCGAGGCTCGCCTTTCGTTTGCCTTTATGTTGAACTCTGCGGTTCAGCGTACAGGTGAACGTGTGACCGCCGAAGAGATTCGGTATGTAGCTTCTGAACTTGAAGATACTTTAGGTGG‑TGTCTACTCTATCCTTTCTCAAGAATTACAATTGCCTCTGGTACGAGTGCTCTTGAAGCAACTACAAGCCACGCAACAGATTCCTGAGTTACCTAAGGAAGCCGTAGAGCCAACCATTAGTACAGGTCTGGAAGCAATTGGTCGAGGACAAGACCTTGATAAGCTGGAGCGGTGTGTCACTGCG  >  NC_001604.1/20987‑21583`

  

Alignment Legend

Aligned base mismatch/match (shaded by quality score): `ATCG/ATCG` `< 3 ≤` `ATCG/ATCG` `< 11 ≤` `ATCG/ATCG` `< 26 ≤` `ATCG/ATCG` `< 36 ≤` `ATCG/ATCG` `< 38 ≤` `ATCG/ATCG`

Unaligned base: `atcg`    Masked matching base: `atcg`    Alignment gap: `‑`    Deleted base: `‑`
